# Small Molecule Fluorescent Ligands for the Atypical Chemokine Receptor 3 (ACKR3)

**DOI:** 10.1021/acsmedchemlett.3c00469

**Published:** 2023-12-08

**Authors:** Sebastian Dekkers, Dehan Comez, Noemi Karsai, Marta Arimont-Segura, Meritxell Canals, Birgit Caspar, Chris de Graaf, Laura E. Kilpatrick, Rob Leurs, Barrie Kellam, Stephen J. Hill, Stephen J. Briddon, Michael J. Stocks

**Affiliations:** † Biodiscovery Institute, School of Pharmacy, University of Nottingham, Nottingham NG7 2RD, United Kingdom; ‡ Centre of Membrane Proteins and Receptors, University of Birmingham and University of Nottingham, The Midlands NG7 2UH, United Kingdom; § Division of Physiology, Pharmacology & Neuroscience, Medical School, University of Nottingham, Nottingham NG7 2UH, U.K.; ∥ Division of Medicinal Chemistry, Amsterdam Institute of Molecular and Life Sciences (AIMMS), Faculty of Science, Vrije Universiteit Amsterdam, De Boelelaan 1108, Amsterdam 1081 HZ, The Netherlands

**Keywords:** Chemokine receptor, ACKR3, CXCR7, Fluorescent probes, BODIPY, NanoBRET

## Abstract

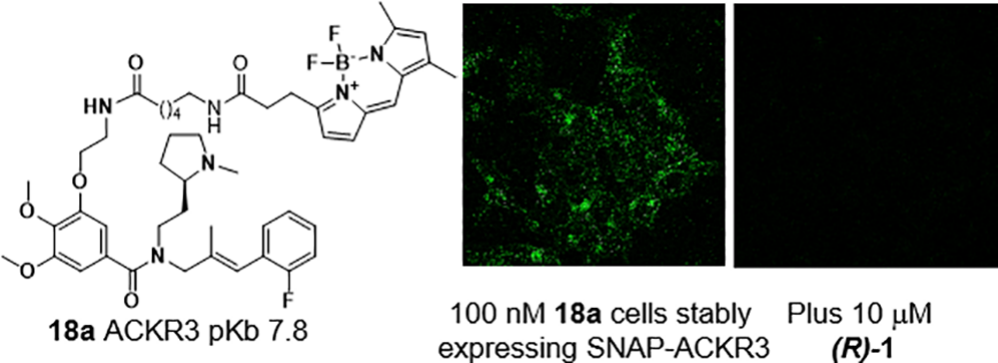

The atypical chemokine receptor 3 (ACKR3) is a receptor that induces cancer progression and metastasis in multiple cell types. Therefore, new chemical tools are required to study the role of ACKR3 in cancer and other diseases. In this study, fluorescent probes, based on a series of small molecule ACKR3 agonists, were synthesized. Three fluorescent probes, which showed specific binding to ACKR3 through a luminescence-based NanoBRET binding assay (p*K*_d_ ranging from 6.8 to 7.8) are disclosed. Due to their high affinity at the ACKR3, we have shown their application in both competition binding experiments and confocal microscopy studies showing the cellular distribution of this receptor.

The atypical chemokine receptor 3 (ACKR3), previously known as CXC-chemokine receptor 7 (CXCR7), is an atypical chemokine receptor belonging to the class A G protein-coupled receptor (GPCR) family. Although the biological role of ACKR3 is not entirely understood, it is reported to function as a scavenger of CXCL12 (C-X-C chemokine 12, also known as SDF-1, stromal cell-derived factor 1) establishing CXCL12 gradients, thereby modulating CXCR4 signaling.^1,2^ It has been postulated to regulate a range of biological functions that occur after binding of the endogenous ligand CXCL12 and subsequent recruitment of the multifunctional intracellular protein β-arrestin, resulting in phosphorylation-dependent receptor internalization without detectable activation of G-proteins.^3^

Expression of ACKR3 on the surface of platelets has been shown to be up-regulated in patients suffering with acute myocardial infarction and subsequent elevation of ACKR3 expression leads to an improvement in recovery.^4,5^ Additionally, increased infarct size and subsequent patient mortality have been observed, where ACKR3 expression has been decreased, signifying the importance of ACKR3 in promoting proliferation and angiogenesis.^6^ ACKR3 is known to be overexpressed in numerous cancer types, indicating its involvement in the modulation of tumor cell proliferation and migration and tumor angiogenesis, contributing to cancer progression and metastasis.^7^ Due to the increasing literature for the role of ACKR3 in disease, several structurally diverse small molecule ACKR3 ligands have been reported (Figure 1).^8,9^

**Figure 1:**
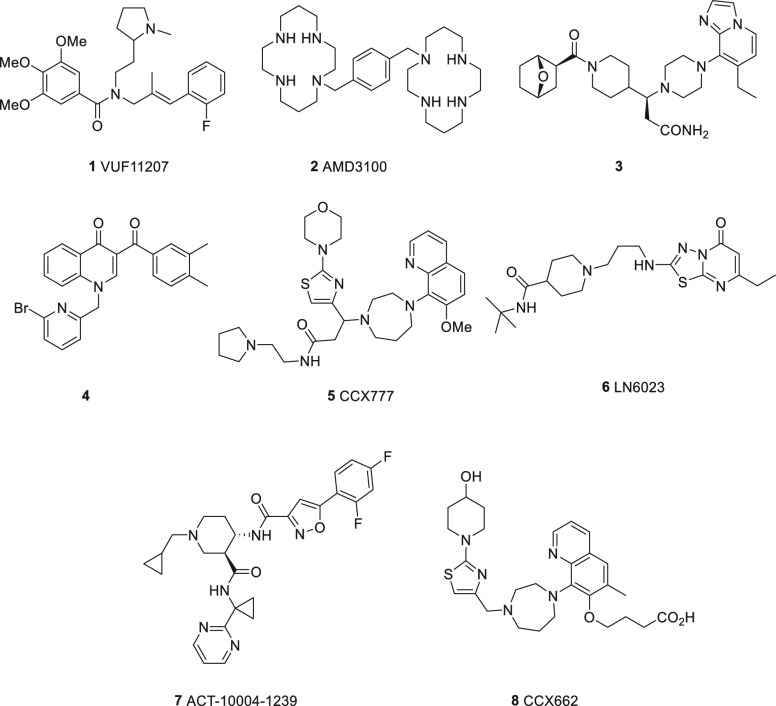
Examples of reported small molecule ACKR3 ligands: **1**,^10^**2**,^11^**3**,^12^**4**,^13^**5**,^14^**6**,^15^**7**,^16^ and **8**.^17^ Details of the affinity or potency of the compounds are shown in the Supporting Information (Table S1).

Currently, the most widely used compound to study ACKR3 function is the endogenous ligand CXCL12. Although human CXCL12 and its radiolabeled and fluorescently labeled versions are available through commercial sources, their arduous synthesis makes them very expensive to employ in both *in vitro* and *in vivo* imaging. Antibodies and nanobodies have also emerged as highly selective tools to study ACKR3^18,19^ but similar to CXCL12, the development of ACKR3-specific antibodies and nanobodies is difficult and time-consuming, making them also very expensive for the medicinal chemist to routinely employ.

Small molecule ligands that selectively target ACKR3 can offer several advantages over chemokines and antibodies as tool compounds to probe receptor function. Though their discovery may be challenging, they are generally more accessible and cheaper for synthetic chemists to make and fluorophore containing analogues offer the potential for detailed visualization of receptor function at a cellular level.^20−23^

We report the synthesis of the first fluorescent ACKR3 probes, based on the receptor agonist VUF11207 (**1**).^10^ An evaluation of the reported structure–activity relationship (SAR) of the small molecule inhibitor, combined with *in silico* docking experiments utilizing the recently disclosed Cryo-EM structure of ACKR3 complexed with the partial agonist **8** CCX662,^17^ informed the synthetic strategy for linker design and fluorophore attachment (Figure 2).

**Figure 2:**
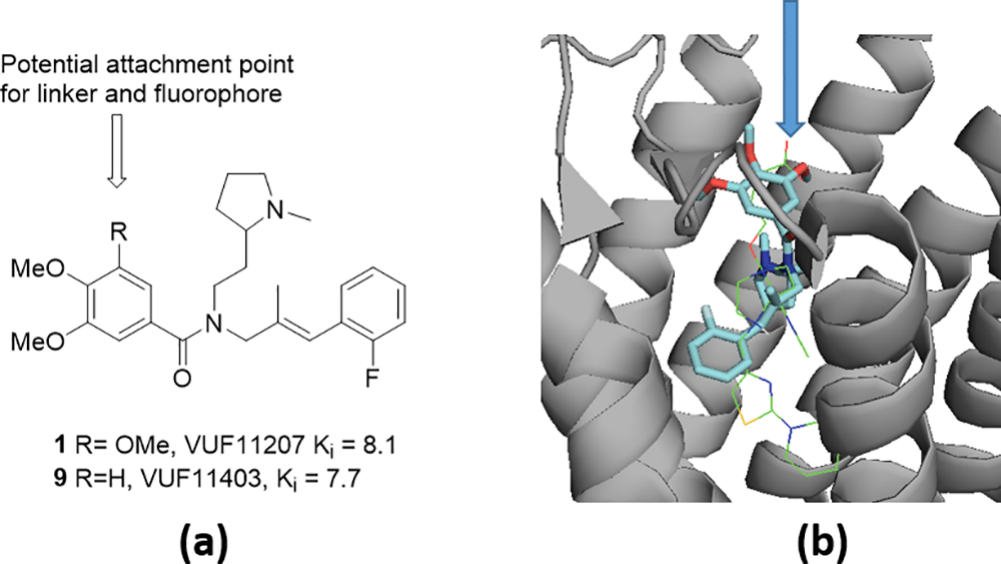
(a) Reported SAR^10^ suggests the highlighted 3-methoxy group present in VUF11207 (**1**) is not essential for ACKR3 binding and can be targeted for linker and fluorophore attachment (b) Docking of VUF11207 (*R*)-**1** into ACKR3 (pdb 7SK9) suggests substitution on the 3-position of the aryl ring would be an appropriate choice for linker and fluorophore attachment. Docking experiments were performed using OEDOCKING Hybrid docking.^24,25^

The resulting fluorescent compounds were characterized in a BRET-based assay, enabled by a NanoLuciferase (NLuc)-ACKR3 construct. The recently developed NanoBRET methodology has allowed characterization of various (fluorescent) probes targeting GPCRs, even when under endogenous promotion.^20,21,26^

The synthesis of fluorescent derivatives of VUF11207 was based on procedures that were described by Wijtmans in the development of VUF11207.^10^ Zarca et al. recently reported on the pharmacological evaluation of the synthesized single enantiomers of VUF11207 (**1**) showing that (*R*)-**1** had a pEC_50_ of 8.3 ± 0.1 compared to (*S*)-**1**, which has a corresponding pEC_50_ of 7.7 ± 0.1 in a [^125^I] CXCL12 displacement assay.^27^

Synthesis started with an aldol reaction between 2-fluorobenzaldehyde and propionaldehyde, which under basic conditions provided (*E*)-3-(2-fluorophenyl)-2-methylacrylaldehyde **10** in excellent yield. A reductive amination with a picoline borane complex and (*R*)-2-(1-methylpyrrolidin-2-yl)ethanamine gave the homochiral precursor **11** in good yield. With this key fragment in hand, we set out to synthesize the various linkers. Here, we chose to develop linkers of three different lengths, with PEG chains ranging from 0 to 2. Commercially available alcohol-carbamates **12a**–**c** were first converted into tosylates using tosyl chloride **13a**–**c**. *O*-Alkylation using methyl 3-hydroxy-4,5-dimethoxybenzoate efficiently installed the linkers on the 3′-position. Hydrolysis of the methyl ester to the benzoic acids **15a**–**c** using lithium hydroxide proceeded with quantitative yields, allowing subsequent peptide coupling with key intermediate **11** to give **16a**–**c** and after *N*-Boc deprotection, the congeners **17a**–**c** were ready for conjugation to commercially available fluorescent dyes (Scheme 1).

**Scheme 1:**
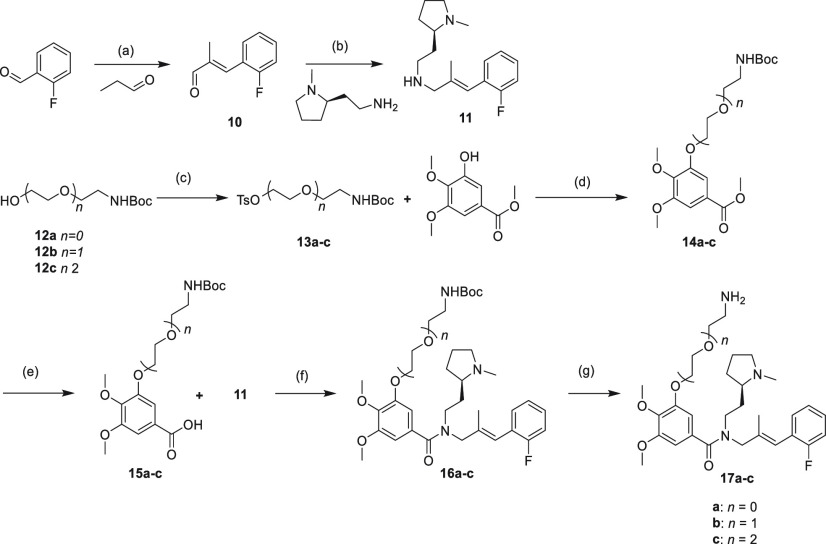
Synthesis of Fluorescent Ligand Precursor’s **17a**–**c** Reagents and conditions: (a) KOH, ethanol, water, stir rt 24 h, 90%; (b) picoline borane complex, methanol, acetic acid, rt, 24 h, 63%; (c) triethylamine, tosyl chloride, DCM, rt, 24 h, 36–40%; (d) cesium carbonate, DMF, rt, 24 h, 63–83%; (e) lithium hydroxide, THF, water, rt, 24 h, quant; (f) HATU, Hunig’s base, DMF, rt, 72–77%; (g) TFA, DCM, rt, quant.

The congeners **17a**–**c** were reacted with the commercial BODIPY FL-X succinimidyl ester to give the corresponding fluorescent ligands **18a**–**c**, after purification by reverse phase HPLC. The fluorescent ligands were prepared in >95% purity as defined through analytical HPLC (Scheme 2).

**Scheme 2:**
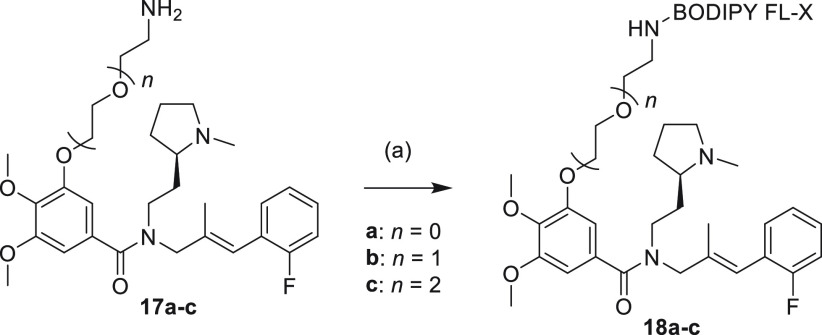
Synthesis of Fluorescent ACKR3 Ligands **18a**–**c** Reagents and conditions: (a) BODIPY FL-X succinimidyl ester, Hunig’s base, acetonitrile, 24 h, extrusion of light (56–77%).

## Pharmacological Evaluation of Fluorescent ACKR3 Antagonists

**Figure 3:**
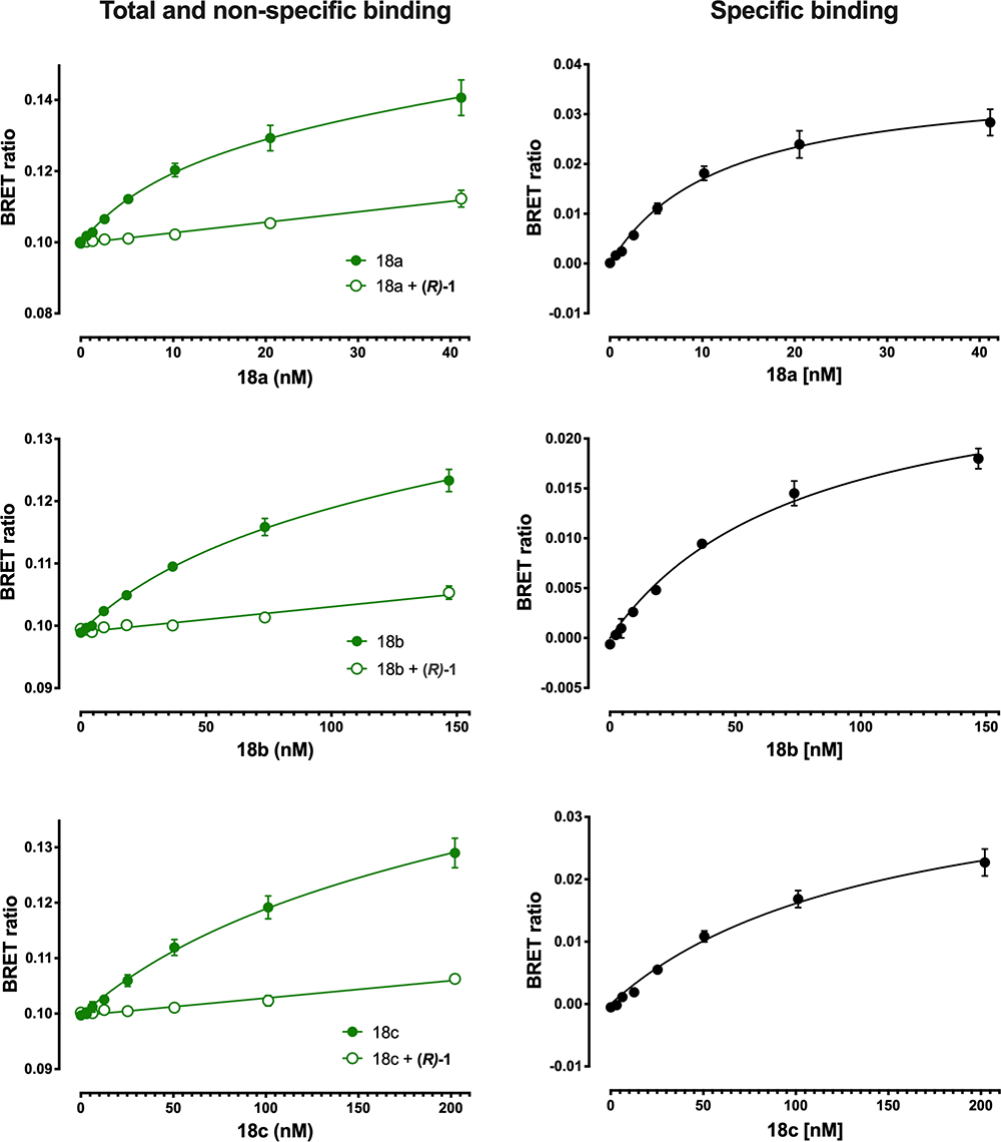
Saturation binding of (*R*)-**1** using **18a**–**c** in HEK293G_NLuc-ACKR3 cells. HEK293G_NLuc-ACKR3 cells were treated with **18a**–**c** in the presence and absence of 10 μM unlabeled (*R*)-**1** full-length *N* terminal NanoLuciferase-ACKR3 stably expressing HEK293 cells. Compounds were added simultaneously and incubated for 60 min at 37 °C in HBSS containing 0.2% BSA. Furimazine (1:400 final dilution) was added and plates incubated for 5 min. Fluorescence and luminescence emissions were measured using a BMG Pherastar FS. The raw BRET ratio was calculated by dividing the fluorescent signal by the bioluminescent signal and specific binding was calculated by deducting nonspecific binding from the total binding values.

The fluorescent conjugates (**18a**–**c**) were evaluated by using a range of pharmacological assays. Initially, saturation binding experiments were used to determine the affinity of the fluorescent conjugates toward the ACKR3 receptor. The fluorescent properties of the compounds allowed detection of the proximity of the fluorescent ligands to an *N*-terminal NanoLuciferase-tagged receptor (NLuc-ACKR3) by means of bioluminescence resonance energy transfer (NanoBRET).^20^ The three fluorescent conjugates produced clear saturable specific binding to the NLuc-ACKR3 receptor that was associated with low levels of nonspecific binding (determined in the presence of unlabeled (*R*)-**1**) resulting in p*K*_d_ values ranging from 6.8 to 7.9 (Figure 3 and Table 1).

**Table 1:** Binding Affinities of **18a**–**c** Determined in HEK293G Cells Expressing NLuc-ACKR3

example	p*K*_d_ (log *M*)^a^	*n*
**18a**	7.89 ± 0.01	4
**18b**	7.09 ± 0.01	4
**18c**	6.82 ± 0.01	4

a p*K*_d_ values were calculated from the negative logarithm of the equilibrium dissociation constant (*K*_d_) determined from saturation-binding experiments using increasing concentrations of labeled ligand in the presence or absence of (*R*)-**1** (10 μM). Data are expressed as mean ± SEM, where each experiment was performed in triplicate.

To further evaluate the use of **18a** in the NanoBRET-ligand binding assay, affinities of ACKR3 ligands **4**, **9**, **19**, and **20** were determined in competition binding experiments (Table 2).
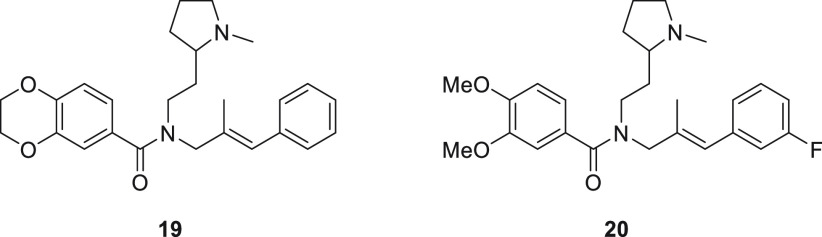


**Table 2:** Binding Affinities of a Series of Known ACKR3 Ligands

example*	p*K*_i_ (log *M*)^a^	*n*
**4**	7.4 ± 0.13	5
**9**	6.6 ± 0.07	6
**19**	5.0 ± 0.10	5
**20**	6.2 ± 0.07	6

a Data are combined mean ± SEM, where each experiment was performed in triplicate.

**Figure 4:**
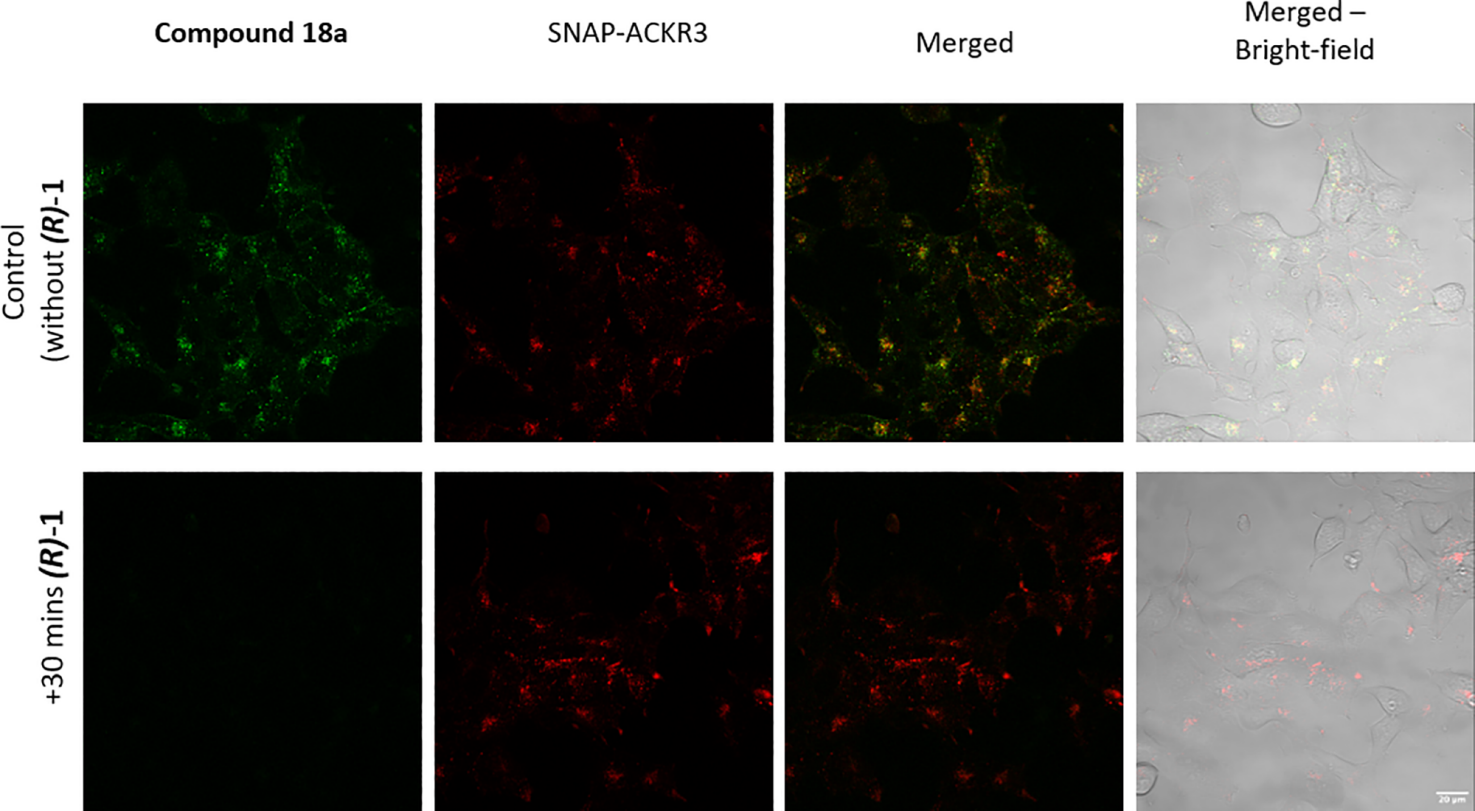
Binding of compound **18a** (100 nM). SNAP-ACKR3 receptor expression (red), Compound **18a** (green) overlay image (third column) with yellow indicating colocalization following incubation for 30 min at 37 °C. Bright-field images of the cells (4th column). Images were taken on a Zeiss LSM710 confocal microscope, 40× 1.2NA water-immersion objective, and representative of four independent experiments.

The availability of high affinity green fluorescent ACKR3 receptor ligands suggested utility for live cell imaging. Confocal microscopy images of fluorescent ligand **18a** incubated with HEK293 cells transiently expressing *N*-terminal SNAPTag-ACKR3 (referred to as SNAP-ACKR3) for 30 min at 37 °C were captured. Under these conditions, SNAP-ACKR3 labeled with the cell impermeable SNAP-AF647 showed a predominantly vesicular intracellular location, with a small amount on the cell membrane (Figure 4, second column). This is consistent with its known high levels of constitutive ACKR3 cycling. Ligand **18a** (100 nM) showed a very similar distribution of mainly intracellular fluorescence, which was colocalized with that of the SNAP-ACKR3 receptor (Figure 4) and may also therefore indicate some ligand induced internalization. Images collected at various time points during incubation of 50 nM **18a** (Supporting Information, Figure S1) indicated that **18a** was initially bound to the cell surface at early time points and then internalized with SNAP-ACKR3. When cells were pretreated with (*R*)-**1**, its level of binding was significantly reduced, suggesting that the majority of observed fluorescence was specific binding of **18a** to the SNAP-ACKR3 receptor.

We have reported the characterization of the first new small molecule-based fluorescent probes for ACKR3. Compounds (**18a**–**c**) retained good affinity toward the ACKR3 receptor, as shown by NanoBRET saturation experiments. We further demonstrated that **18a** is a useful screening tool for discovering new ACKR3 agonists. Compound **18a** displayed good signal-to-noise in NanoBRET competition-binding experiments and was displaced by the established small molecule agonist (*R*)-**1**, close analogues, and a structurally diverse agonist **4**. The fluorescent ACKR3 ligands (**18a**–**c**) can be used in live cell confocal microscopy experiments and in combination with the NanoBRET approach may shed further light on ACKR3 function and its participation in pathophysiological conditions.

## Supporting Information Available

The Supporting Information is available free of charge on the ACS Publications Web site. The Supporting Information is available free of charge at https://pubs.acs.org/doi/10.1021/acsmedchemlett.3c00469.

Method for compound preparation, LCMS traces of **18a**–**c**, pharmacological methods, time course confocal imaging of **18a**, pharmacological data for selected literature compounds (PDF)

## Abbreviations

ACKR3: atypical chemokine receptor 3
BODIPY: boron dipyrromethene
BRET: bioluminescence resonance energy transfer
BSA: bovine serum albumin
CXCL12: C X-C chemokine ligand type 12
CXCR4: CXC-chemokine receptor type 4
CXCR7 CXC-chemokine receptor 7: DMF, dimethylformamide
HEK293G: human embryonic kidney cells expressing a GloSensor biosensor
LC/MS: liquid chromatography/mass spectrometry
NLuc: NanoLuciferase
NMR: nuclear magnetic resonance
PBS: phosphate-buffered saline
RP-HPLC: reverse phase high performance liquid chromatography
SAR: structure–activity relationship
SDF: stromal cell-derived factor 1
TOF ES+: positive electrospray ionization time-of-flight
