# Supplementary material for: Small Molecule Fluorescent Ligands for the Atypical Chemokine Receptor 3 (ACKR3)
Source: ACS Med Chem Lett. 2023 Dec 8;15(1):143–8. doi: 10.1021/acsmedchemlett.3c00469 (PMC10788940; doi:10.1021/acsmedchemlett.3c00469)
Supplement: Supplementary file 1 — ml3c00469_si_001.pdf [file ml3c00469_si_001.pdf]

Supporting Information

Small molecule fluorescent ligands for the atypical chemokine receptor 3 (ACKR3).

*Sebastian Dekkers,[1] Dehan Comez,[2,3] Noemi Karsai,[2,3] Marta Arimont-Segura,[4] Meritxell Canals,[2,3] Birgit Caspar,[2,3] Chris de Graaf,[4] Laura E. Kilpatrick,[1,2] Rob Leurs,[4] Barrie Kellam,[1,2] Stephen J. Hill,[2,3] Stephen J. Briddon,[2,3] Michael J. Stocks[1]**

[1]Biodiscovery Institute, School of Pharmacy, University of Nottingham, Nottingham, NG7 2RD, United Kingdom

[2]Centre of Membrane Proteins and Receptors, University of Birmingham and University of Nottingham, The Midlands, NG7 2UH, United Kingdom.

[3]Division of Physiology, Pharmacology & Neuroscience, Medical School, University of Nottingham, Nottingham, NG7 2UH, UK.

[4]Division of Medicinal Chemistry, Amsterdam Institute of Molecular and Life Sciences (AIMMS), Faculty of Science, Vrije Universiteit Amsterdam, De Boelelaan 1108, Amsterdam 1081 HZ, The Netherlands.

*Corresponding Author: michael.stocks@nottingham.ac.uk

Contents: **Page**

Synthetic chemistry methods S2

Pharmacology: Material and Methods S10

Figure S1: Time-dependent binding of Compound **18a** S13

Table S1: Pharmacological activities of known ACKR3 ligands S14

General Experimental procedures

Synthetic chemistry - no unexpected or unusually high safety hazards were encountered

Materials and Methods. HPLC grade and analytical grade chemicals and solvents were purchased from the standard suppliers and were used without further purification. BODIPY fluorophores were obtained from Molecular Probes (Fischer Scientific, U.K.). Sigma Aldrich supplied high-grade silica, 60 Å, 230-400 mesh, for flash chromatography, and deuterated solvents (Chloroform-d, Methanol-d4, DMSO-d6) were purchased from Sigma Aldrich. Reactions were monitored by thin-layer chromatography (TLC) on commercially available silica pre-coated aluminum-backed plates (Merck Kieselgel 60 F254). Visualization was under UV light (254 nm and 366 nm), and where necessary staining with ninhydrin or potassium permanganate dips. NMR spectra were recorded with a Bruker AV(III) 400 NMR spectrometer. ^{1}H NMR was recorded at 400.13 MHz, and 13C NMR was recorded at 101.6 MHz. Deuterated solvents used for the preparation of NMR samples were CDCl3, MeOD-d_4, or DMSO-d_6. Chemical shifts (δ) are reported in ppm with reference to the chemical shift of the deuterated solvent. Coupling constants (J) are recorded in hertz, and the signal multiplicities are described by the following: s, singlet; d, doublet; t, triplet; q, quartet; brs, broad singlet; m, multiplet; dd, doublet of doublets; ddd, double doublet of doublets; dt, doublet of triplets; p, pentet. NMR data was processed using MestReNova version 10.0.2. For the analysis of reaction mixtures and isolated compounds a Shimadzu UFLCXR HPLC was used, equipped with a Biosystems MDS SCIEX API2000 ESI+ MS. The column was a Gemini 3 µm C18 110 Å, LC column 50 x 2 nm. As eluent a mixture was used of MeCN and H_2O, containing 0.1% formic acid. Samples were run using a gradient of 1:19 v/v to 19:1 v/v over either 5 or 15 minutes, with a flowrate of 0.5 mL/min. UV absorption was detected at 254 nm and 220 nm. Preparative RP-HPLC was performed on a Waters 2767 sample manager coupled to Waters 2525 binary-gradient module and a Waters 2457 dual-wavelength absorbance detector. The column used was a Phenomenex Gemini-NX (5 µm, 110 Å, C18, 150 × 21 mm) at ambient temperature. The flow rate was 25 mL/min, and UV detection was at 254 nm. Mobile phases were solvent A, 0.1% TFA in water, and solvent B, acetonitrile, degassed by helium bubble and sonication, respectively. HRMS was done on a Bruker microTOF II mass spectrometer using electrospray ionization (ESI-TOF) operating in the positive mode. Adducts within errors of ±10 ppm were reported

(R,E)-3-(2-fluorophenyl)-2-methyl-N-(2-(1-methylpyrrolidin-2-yl)ethyl)prop-2-en-1-amine (11):

A round bottom flask containing 10 mL MeOH was charged with *(R)*-2-(1-methylpyrrolidin-2-yl)ethanamine dihydrochloride (0.5 g, 2.49 mmol) and triethylamine (0.7 mL, 2 eq.). This mixture was stirred at room temperature for 30 minutes before addition of 2.5 mL glacial AcOH and MeOH, reaching a final volume of 25 mL. Aldehyde fragment 10 (0.408g, 1 eq.) and Na_2SO_4 (150 mg, 1.0 mmol) were added and the mixture was left to stir at room temperature overnight. Finally, 2-Picoline borane complex (0.266 g, 1 eq.) was added and the reaction was left to stir until completion (24 hours). The reaction mixture was concentrated in vacuo and the residue was taken up in H2O. The aqueous was washed with DCM (3x 100 mL), after which it was basified to pH 14.0 using concentrated NaOH. This was then extracted with DCM (5x 100 mL), the organics combined, dried over $MgSO_4$ and concentrated *in vacuo*. Column purification using 2.5-7% 7N NH_3 in MeOH in DCM yielded our desired fragment as a colourless oil. Yield: 0.435g (1.57 mmol, 63%).

LCMS analytical calc. for $C_{17}H_{25}FN_2$ = 276.20, found 277.2 [M+H].

^{1}H NMR (400 MHz, $CDCl_3$) δ 7.16 (t, J = 7.6 Hz, 1H), 7.12 – 7.02 (m, 1H), 7.01 – 6.86 (m, 2H), 6.32 (s, 1H), 3.24 (s, 2H), 2.95 (ddd, J = 9.6 Hz, J = 7.8 Hz, J = 2.1 Hz, 1H), 2.70 – 2.47 (m, 2H), 2.22 (s, 3H), 2.07 – 1.95 (m, 2H), 1.90 – 1.75 (m, 2H), 1.71 (s, 3H), 1.67 – 1.49 (m, 2H), 1.48 – 1.30 (m, 2H).

^{13}C NMR (101 MHz, $CDCl_3$) δ 161.25, 158.80, 139.47, 130.61, 130.58, 127.91, 127.83, 125.65, 125.51, 123.37, 123.34, 118.05, 118.03, 115.26, 115.04, 64.54, 57.75, 57.14, 57.12, 46.50, 40.39, 40.37, 33.92, 30.61, 21.90, 16.59, 16.57.

Representative experimental for preparation of intermediates 13a-c

2-((tert-Butoxycarbonyl)amino)ethyl 4-methylbenzenesulfonate (**13a**):

N-Boc-ethanolamine (1 mL, 6.46 mmol) was taken up in 20 mL DCM, and to it was added 1.8 mL (2 eq.) TEA. This mixture was stirred for 5 minutes at room temperature, after which it was cooled to 0 °C. Tosyl chloride (1.23 g, 1 eq.) was dissolved in 10 mL DCM and added to the reaction mixture in a dropwise fashion. When the addition was completed the reaction mixture was allowed to slowly reach room temperature, at which it was maintained overnight. Upon completion (TLC), the solvents were evaporated in vacuo and the product was purified by column chromatography using 20-100% EtOAc in hexane as eluent. The product was isolated as an off white solid. Yield: 0.8g (2.5 mmol, 40%).

LCMS analytical calc. for $C_{14}H_{21}NO_5S$ = 315.11, found 316.1 [M+H]. ^{1}H NMR (400 MHz, $CDCl_3$) δ 7.78 (d, J = 8.0 Hz, 2H), 7.35 (d, J = 8.0 Hz, 2H), 4.93 (s, NH, 1H), 4.06 (t, J = 5.2 Hz, 2H), 3.37 (q, J = 5.2 Hz, 2H), 2.44 (s, 3H), 1.40 (s, 9H). ^{13}C NMR (101 MHz, $CDCl_3$) δ 155.62, 145.02, 132.64, 129.95, 127.91, 79.73, 69.44, 39.73, 28.28, 21.63.

General procedures for the O-alkylation of 4,5-dimethyoxy-3-hydroxybenzoate (compounds 1**4a-c**):

To a round bottom flask containing 15 mL DMF were added 4,5-dimethyoxy-3-hydroxybenzoate (1 eq.), the appropriate tosylated alcohol **13a-c** (1 eq.), and $CsCO_3$ (3 eq.). The reaction was carried out at 60 °C until LCMS indicated completion. The reaction mixture was then poured onto brine and extracted with EtOAc (3x 50mL). The organics were combined, dried over $NaSO_4$ and concentrated *in vacuo*. The products were purified by column chromatography using 50% EtOAc in hexane as eluent.

*Methyl 3-(2-((tert-butoxycarbonyl)amino)ethoxy)-4,5-dimethoxybenzoate (**14a**):*

Prepared using 0.343g (1.09 mmol) 2-((tert-butoxy carbonyl)amino)ethyl 4-methylbenzenesulfonate (**13a**). Yield: 0.244g (0.69 mmol, 63%, colourless oil).

LCMS analytical calc. for $C_7H_{25}NO_7$ = 355.16, found 256.1 LCMS: 356.1 [M+H]. ^{1}H NMR (400 MHz, $CDCl_3$) δ 7.30 – 7.21 (m, 2H), 5.15 (br s, 1H), 4.07 (t, J = 5.2 Hz, 2H), 3.86 (s, 6H), 3.86 (s, 3H), 3.52 (q, J = 5.5 Hz, 2H), 1.40 (s, 9H). ^{13}C NMR (101 MHz, $CDCl_3$) δ 166.45, 155.87, 153.03, 151.86, 142.69, 125.15, 108.77, 107.17, 79.37, 68.81, 60.89, 56.16, 52.15, 40.07, 28.33.

*Methyl 3-(2-(2-((tert-butoxycarbonyl)amino)ethoxy)ethoxy)-4,5-dimethoxy benzoate (**14b**):*

Prepared using 0.12 g (0.33 mmol) 2-(2-((tert-butoxycarbonyl)amino)ethoxy)ethyl 4-methylbenzene sulfonate (**13b**). Yield: 0.104g (0.26 mmol, 80%, colourless oil).

LCMS analytical calc. for $C_{19}H_{29}NO_8$ = 399.2, found 400.4 [M+H]. ^{1}H NMR (400 MHz, $CDCl_3$) δ 7.31 (dd, J = 15.0 Hz, J = 1.9 Hz, 2H), 5.02 (br s, 1H), 4.27 – 4.16 (m, 2H), 3.91 (s, 3H), 3.89 (s, 6H), 3.86 – 3.78 (m, 2H), 3.60 (t, J = 5.1 Hz, 2H), 3.32 (q, J = 5.4 Hz, 2H), 1.41 (s, 9H). ^{13}C NMR (101 MHz, $CDCl_3$) δ 166.60, 155.95, 153.02, 152.07, 142.72, 124.98, 109.99, 108.71, 107.04, 79.15, 70.35, 69.43, 68.76, 60.86, 56.22, 52.19, 40.42, 28.36.

(14c):

Prepared using 0.146g (0.36 mmol) 2,2-dimethyl-4-oxo-3,8,11-trioxa-5-azatridecan-13-yl 4-methylbenzenesulfonate (**13c**). Yield: 0.133g (0.3 mmol, 83%, colourless oil).

LCMS analytical calc. for $C_{21}H_{33}NO_9$ = 433.22, found 444.2 [M+H].^{1}H NMR (400 MHz, $CDCl_3$) δ 7.30 (dd, *J* = 10.1 Hz, *J* = 6.5 Hz, 2H), 5.05 (br s, 1H), 4.22 (t, *J* = 5.6 Hz, 2H), 3.94 – 3.86 (m, 11H), 3.77 – 3.68 (m, 2H), 3.67 – 3.58 (m, 2H), 3.54 (t, *J* = 5.2 Hz, 2H), 3.30 (q, *J* = 5.4 Hz, 2H), 1.42 (s, 9H). ^{13}C NMR (101 MHz, $CDCl_3$) δ 166.59, 155.96, 153.00, 152.06, 142.74, 124.99, 108.69, 107.01, 79.11, 70.75, 70.31, 70.27, 69.60, 68.81, 60.86, 56.22, 52.18, 40.36, 28.38.

General procedure for the ester hydrolysis of methyl esters 14a-c:

To a round bottom flask containing a mixture of THF and H2O (1:1) were added the appropriate methyl ester intermediate **13a-c** (1 eq.) and lithium hydroxide monohydrate (3 eq.). This solution was stirred at room temperature until LCMS and TLC indicated full conversion. The THF was removed *in vacuo* and H_2O was added to dilute the reaction mixture. This was then washed with EtOAc (2x 20 mL), after which the aqueous was acidified using 2N aq. HCl. The product was extracted with EtOAc (3x 30 mL), the organics were combined and dried over $MgSO_4$.

3-(2-((tert-butoxycarbonyl)amino)ethoxy)-4,5-dimethoxybenzoic acid **(15a):**

Prepared using 0.1g (0.28 mmol) of methyl ester **14a**. Yield: 95 mg (0.28 mmol, quant., colourless oil).

LCMS analytical calc. for $C_{16}H_{23}NO_7$ = 341.15, found 342.1 [M+H]. ^{1}H NMR (400 MHz, $CDCl_3$) δ 10.56 (br s, 1H), 7.37 (s, 2H), 5.18 (br s, 1H), 4.13 (t, *J* = 5.2 Hz, 2H), 4.03 – 3.79 (m, 6H), 3.59 (q, *J* = 5.5 Hz, 2H), 1.46 (s, 9H). ^{13}C NMR (101 MHz, $CDCl_3$) δ 170.76, 156.06, 153.08, 151.91, 143.30, 124.57, 109.37, 107.74, 79.71, 68.90, 60.99, 56.22, 40.12, 28.38.

3-(2-(2-((tert-butoxycarbonyl)amino)ethoxy)ethoxy)-4,5-dimethoxybenzoic acid **15b):**

Prepared using 0.104g (0.26 mmol) of methyl ester **34b**. Yield: 0.1 g (0.26 mmol, quant., brownish oil).

LCMS analytical calc. for $C_{18}H_{27}NO_8$ = 385.17, found 386.4 [M+H]. ^{1}H NMR (400 MHz, $CDCl_3$) δ 10.19 (br s, 1H), 7.37 (d, *J* = 15.2 Hz, 2H), 5.11 (br s, 1H), 4.22 (t, *J* = 4.5 Hz, 2H), 4.05 – 3.82 (m, CH_2, 8H), 3.63 (t, *J* = 5.1 Hz, 2H), 3.35 (q, *J* = 5.4 Hz, 2H), 1.43 (s, 9H). ^{13}C NMR (101 MHz, $CDCl_3$) δ 170.77, 156.14, 153.02, 152.06, 143.27, 124.37, 109.21, 107.56, 79.34, 70.35, 69.44, 68.74, 60.90, 56.23, 40.42, 28.37.

3-((2,2-Dimethyl-4-oxo-3,8,11-trioxa-5-azatridecan-13-yl)oxy)-4,5-dimethoxybenzoic acid **(15c):**

Prepared using 0.133g (0.3 mmol) of methyl ester **14c**. Yield: 0.124 g (0.3 mmol, quant., colourless oil).

LCMS analytical calc. for $C_{20}H_{31}NO_9$ = 429.20, found 430.1 [M+H]. ^{1}H NMR (400 MHz, $CDCl_3$) δ 10.24 (br s, 1H), 7.36 (dd, *J* = 14.4 Hz, *J* = 1.8 Hz, 2H), 5.13 (br s, 1H), 4.24 (t, *J* = 4.8 Hz, 2H), 3.92 (d, *J* = 9.9 Hz, 8H), 3.74 (dd, *J* = 5.8 Hz, *J* = 3.4 Hz, 2H), 3.65 (dd, *J* = 5.8 Hz, *J* = 3.4 Hz, 2H), 3.56 (t, *J* = 5.2 Hz, 2H), 3.32 (q, *J* = 5.5 Hz, 2H), 1.43 (s, 9H). ^{13}C NMR (101 MHz, $CDCl_3$) δ 177.06, 156.17, 153.01, 152.08, 143.34, 124.25, 109.29, 107.51, 79.35, 70.74, 70.28, 69.65, 68.85, 60.91, 56.23, 40.35, 28.38, 14.17.

General procedure for the peptide coupling between amine fragment 11 and compounds 15a-c:

To a round bottom flask containing DMF were added **11** (1 eq.), corresponding benzoic acid **14a-c** (1 eq.), HATU (1 eq.), and DIPEA (2 eq.). This mixture was stirred at room temperature for 2 hours, or until completion (LCMS). The solvent was evaporated *in vacuo* and the crude was purified by column chromatography using 4% 7N NH_3 in MeOH in DCM as eluent.

Tert-butyl (*R*,*E*)-(2-(5-((3-(2-fluorophenyl)-2-methylallyl)(2-(1-methylpyrrolidin-2-yl)ethyl)carbamoyl)-2,3-dimethoxyphenoxy)ethyl)carbamate **(16a):**

Prepared using 40 mg (0.11 mmol) of **11**. Yield: 48 mg (0.08 mmol, 72%, brownish solid). LCMS analytical calc. for $C_{33}H_{46}FN_3O_6$ = 599.34, found 600.5 [M+H]. ^{1}H NMR (400 MHz, $CDCl_3$) δ 7.28 – 7.21 (m, 2H), 7.13 (td, *J* = 7.5 Hz, *J* = 1.2 Hz, 1H), 7.07 (t, *J* = 10.1 Hz, *J* = 8.2 Hz, 1H), 6.83 – 6.62 (m, 2H), 6.45 (s, 1H), 5.09 (br s, 1H), 4.13 – 3.92 (m, 4H), 3.86 (s, 6H), 3.59 – 3.47 (m, 2H), 3.16 – 2.92 (m, 1H), 2.37 (s, 2H), 2.27 – 1.92 (m, 6H), 1.90 – 1.52 (m, 6H), 1.49-1.42 (m, 11H). ^{13}C NMR (101 MHz, $CDCl_3$) δ 171.43, 161.31, 158.86, 155.85, 153.46, 152.17, 139.63, 136.19, 131.86, 130.47, 128.68, 123.72, 119.37, 115.58, 115.36, 105.80, 104.41, 79.46, 68.89, 64.21, 60.96, 57.14, 56.19, 40.50, 40.06, 38.60, 30.94, 30.55, 28.38, 21.98, 16.05.

Tert-butyl (*R*,*E*)-(2-(2-(5-((3-(2-fluorophenyl)-2-methylallyl)(2-(1-methylpyrrolidin-2-yl)ethyl)carbamoyl)-2,3-dimethoxyphenoxy)ethoxy)ethyl)carbamate **(16b):**

Prepared using 40 mg (0.11 mmol) of **11**. Yield: 54 mg (0.08 mmol, 72%, brownish solid). LCMS analytical calc. for $C_{35}H_{50}FN_3O_7$ = 643.36, found 644.6 [M+H]. ^{1}H NMR (400 MHz, $CDCl_3$) δ 7.26 – 7.21 (m, 2H), 7.13 (t, *J* = 7.5 Hz, 1H), 7.07 (t, *J* = 8.2 Hz, 1H), 6.76 – 6.62 (m, 2H), 6.45 (s, 1H), 5.04 (br s, 1H), 4.39-4.25 (m, 1H), 4.12 (s, 2H), 4.04-3.96 (m, 1H), 3.89-3.76 (m, 8H), 3.59 (q, *J* = 5.5 Hz, 2H), 3.31 (q, *J* = 5.4 Hz, 2H), 3.09-3.00 (m, 1H), 2.35-1.95 (m, 7H), 1.84-1.58 (m, 7H), 1.49-1.42 (m, 11H). ^{13}C NMR (101 MHz, $CDCl_3$) δ 171.53, 162.49, 161.31, 158.85, 155.96, 153.34, 152.32, 139.58, 136.21, 130.46, 130.43, 128.68, 123.70, 115.55, 115.33, 105.61, 104.17, 79.15, 70.35, 69.35, 64.15, 60.84, 57.16, 56.19, 53.43, 42.91, 40.41, 38.59, 36.45, 28.39, 21.99, 16.06.

Tert-butyl (R,E)-(2-(2-(2-(5-((3-(2-fluorophenyl)-2-methylallyl)(2-(1-methylpyrro-lidin-2-yl)ethyl)carbamoyl)-2,3-dimethoxyphenoxy)ethoxy)ethoxy)ethyl)-carbamate **(16c):**

Prepared using 30 mg (0.11 mmol) of **11**. Yield: 58 mg (0.08 mmol, 77%, colourless oil). LCMS analytical calc. for $C_{37}H_{54}FN_3O_8$ = 687.39, found 688.4 [M+H]. ^{1}H NMR (400 MHz, $CDCl_3$) δ 7.39 – 7.17 (m, 2H), 7.11 (td, *J* = 7.5 Hz, *J* = 1.2 Hz, 1H), 7.08 – 7.00 (m, 1H), 6.75 – 6.61 (m, 2H), 6.44 (s, 1H), 5.06 (br s, 1H), 4.47 – 4.21 (m, 4H), 4.22 – 4.09 (m, 2H), 4.06 – 3.93 (m, 1H), 3.93 – 3.77 (m, 8H), 3.69 (t, *J* = 4.6 Hz, 2H), 3.61 (dd, *J* = 5.8 Hz, *J* = 3.5 Hz, 2H), 3.52 (t, *J* = 5.2 Hz, 2H), 3.47 – 3.35 (m, 1H), 3.33 – 3.25 (m, 2H), 3.12 – 2.91 (m, 1H), 2.47 – 2.25 (m, 2H), 2.25 – 1.91 (m, 2H), 1.88-1.55 (m, 8H), 1.42 (s, 9H). ^{13}C NMR (101 MHz, $CDCl_3$) δ 171.53, 161.30, 158.85, 155.97, 153.35, 152.41, 139.58, 136.21, 130.46, 128.67,

123.70, 119.35, 115.54, 115.33, 105.62, 104.15, 79.11, 70.72, 70.32, 70.27, 69.60, 68.82, 60.83, 57.15, 56.26, 56.18, 40.36, 28.40, 21.98, 16.04.

General procedure for the *N*-Boc deprotection of intermediates 16a-c:

The appropriate Boc-protected intermediate **16a-c** was taken up in a 4:1 mixture of DCM and TFA, and was stirred at room temperature until completion (LCMS). The reaction mixture was concentrated *in vacuo* and residual TFA was removed by co-evaporation with toluene. The residue was then taken up in H_2O and washed with EtOAc (2x 20 mL), after which the aqueous was basified to pH 14.0 using concentrated aq. NaOH. The product was then extracted using DCM (5x 20 mL), the combined organics dried over Na_2SO_4, and finally the solvents were removed *in vacuo* to afford our desired congeners as a freebase. For storage purposes these compounds were kept as diHCl salts, by stirring the compounds in 4N HCl in dioxane for 5 minutes, and evaporation of the solvents.

(R,E)-3-(2-aminoethoxy)-N-(3-(2-fluorophenyl)-2-methylallyl)-4,5-dimethoxy-N-(2-(1-methylpyrrolidin-2-yl)ethyl)benzamide **(17a):**

Prepared using 46 mg (0.08 mmol) compound **16a**. Yield: 38 mg (0.08 mmol, quant., colourless oil).

LCMS analytical calc. for $C_{28}H_{38}FN_3O_4$ = 499.28, found 500.3 [M+H]. ^{1}H NMR (400 MHz, DMSO-d_6) δ 11.04 (s, 1H), 8.39 (s, 3H), 7.46 – 7.29 (m, 2H), 7.28 – 7.16 (m, 2H), 6.91 – 6.71 (m, 2H), 6.56 – 6.32 (m, 1H), 4.21-4.09 (m, 2H), 4.08-3.92 (m, 2H), 3.87-3.82 (m, 1H), 3.75 (s, 6H), 3.55-3.44 (m, 1H), 3.33-3.12 (m, 3H), 3.08-3.03 (m, 1H), 2.80-2.71 (m, 5H), 2.02-1.70 (m, 5H), 1.65 (s, 3H). ^{13}C NMR (101 MHz, DMSO-d_6) δ 170.87, 161.08, 158.65, 153.32, 151.80, 137.28, 131.19, 131.16, 129.43, 124.64, 118.77, 115.93, 115.72, 106.96, 105.14, 66.48, 65.38, 60.94, 56.40, 55.23, 29.66, 27.89, 21.51, 16.33, 15.64.

(*R*,*E*)-3-(2-(2-aminoethoxy)ethoxy)-*N*-(3-(2-fluorophenyl)-2-methylallyl)-4,5-dimethoxy-*N*-(2-(1-methylpyrrolidin-2-yl)ethyl)benzamide **(17b):**

Prepared using 54 mg (0.08 mmol) compound **16b**. Yield: 46 mg (0.08 mmol, quant., brownish oil).

LCMS analytical calc. for $C_{30}H_{42}FN_3O_5$ = 543.31, found 544.3 [M+H]. ^{1}H NMR (400 MHz, DMSO-d_6) δ 11.51 – 11.05 (m, 1H), 8.58 – 8.02 (m, 3H), 7.47 – 7.29 (m, 2H), 7.23 (dt, *J* = 10.4 Hz, *J* = 6.8 Hz, 2H), 6.95 – 6.55 (m, 2H), 6.53 – 6.34 (m, 1H), 4.41 – 4.07 (m, 3H), 3.75 (m, 10H), 3.53-3.50 (m, 3H), 3.28-3.24 (m, 1H), 2.97 (q, *J* = 5.5 Hz, 2H), 2.79-2.70 (m, 3H), 2.32 (d, *J* = 10.4 Hz, 1H), 1.99 (s, 3H), 1.88 – 1.72 (m, 3H), 1.66 (s, 3H). ^{13}C NMR (101 MHz, DMSO-d_6) δ 161.08, 158.65, 153.19, 152.31, 137.40, 131.18, 131.14, 129.41, 124.66, 115.93, 115.71, 105.79, 104.53, 69.31, 68.65, 67.15, 66.26, 60.62, 56.36, 55.21, 49.03, 42.78, 29.69, 27.90, 21.51, 16.35, 14.55.

(*R*,*E*)-3-(2-(2-(2-aminoethoxy)ethoxy)ethoxy)-*N*-(3-(2-fluorophenyl)-2-methylallyl)-4,5-dimethoxy-*N*-(2-(1-methylpyrrolidin-2-yl)ethyl)benzamide **(17c):**

Prepared using 58 mg (0.085 mmol) compound **16c**. Yield: 50 mg (0.085 mmol, quant., colourless oil).

LCMS analytical calc. for $C_{32}H_{46}FN_3O_6$ = 587.34, found 588.3 [M+H]. ^{1}H NMR (400 MHz, DMSO-d_6) δ 11.61 – 10.84 (m, 1H), 8.15 (s, 3H), 7.42 – 7.32 (m, 2H), 7.30 – 7.16 (m, 2H), 6.85 – 6.65 (m, 2H), 6.53 – 6.30 (m, 1H), 4.23 – 4.00 (m, 3H), 3.90 – 3.69 (m, 10H), 3.69 –

3.42 (m, 7H), 3.34 – 3.17 (m, 1H), 3.08-2.88 (m, 3H), 2.87 – 2.64 (m, 4H), 2.41 – 2.22 (m, 2H), 2.12 – 1.55 (m, 6H). ^{13}C NMR (101 MHz, DMSO-d_6) δ 171.02, 161.08, 158.65, 153.19, 152.35, 137.43, 131.18, 129.41, 124.67, 124.63, 118.43, 115.93, 115.71, 105.72, 104.53, 70.22, 70.14, 69.38, 68.76, 67.10, 66.28, 65.38, 60.56, 56.36, 56.03, 55.23, 42.81, 29.67, 27.92, 21.52, 16.36, 15.64.

General procedure for the peptide coupling between 17a-c and BODIPY FL-succinimidyl ester:

To a small round bottom flask was added 1 mL DMF, BODIPY FL-X NHS ester (2 eq.), HATU (1 eq.), *N*-hydroxysuccinimide (1.3 eq.), and DIPEA (6 eq.). This mixture was stirred in the dark at room temperature for 1 hour, after which the corresponding congener (1 eq.) was added. This final reaction mixture was left to stir in the dark until completion, after which the reaction mixture was concentrated *in vacuo*, and the crude purified by RP-HPLC (5-95% solvent B, 30 min).

(R,E)-3-(2-(6-(3-(5,5-difluoro-7,9-dimethyl-5H-4λ^4,5λ^4-dipyrrolo[1,2-c:2',1'-f][1,3,2] diazaborinin-2-yl)propanamido)hexanamido)ethoxy)-N-(3-(2-fluorophenyl)-2-methylallyl)-4,5-dimethoxy-N-(2-(1-methylpyrrolidin-2-yl)ethyl)benzamide **(18a):**

Prepared using BODIPY FL-X NHS ester (1 mg, 2.0 pmol) and compound **17a** (2 mg, 2 eq.). LCMS showed complete conversion of the starting materials to the product after 5 hours (mass found: 887.5; M+H$^+$). Yield: 1.0 mg (56%). Purity of the compound was confirmed to be >95% by LCMS using a long method run.

HRMS calculated for $C_{48}H_{62}BF_3N_6O_6$: 887.4854 [M+H], found: 887.4860 [M+H].

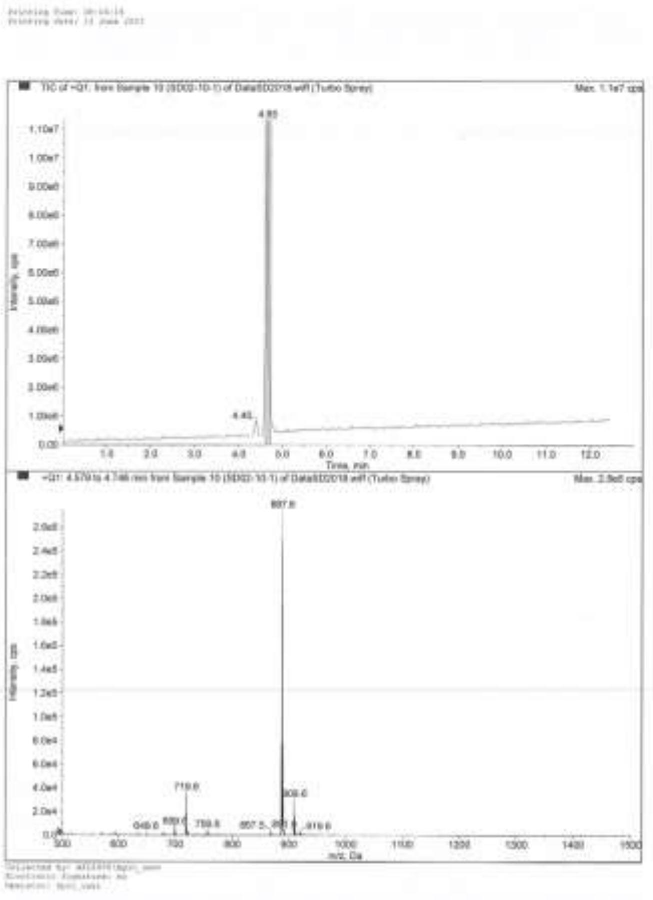

(*R*,*E*)-3-(2-(2-(6-(3-(5,5-difluoro-7,9-dimethyl-5*H*-4l4,5l4-dipyrrolo[1,2-*c*:2',1'-*f*][1,3,2]diazaborinin-2-yl)propanamido)hexanamido)ethoxy)ethoxy)-*N*-(3-(2-fluorophenyl)-2-methylallyl)-4,5-dimethoxy-*N*-(2-(1-methylpyrrolidin-2-yl)ethyl)benzamide **(18b):**

Prepared using BODIPY FL-X NHS ester (1 mg, 2.0 pmol) and compound **17b** (2 mg, 2 eq.). LCMS showed complete conversion of the starting materials to the product after stirring overnight (mass found: 931.5; M+H$^+$). Yield: 1.3 mg (70%). Purity of the compound was confirmed to be >95% by LCMS using a long method run.

HRMS calculated for $C_{50}H_{65}BF_3N_6O_7$: 931.5116 [M+H], found: 931.5125 [M+H].

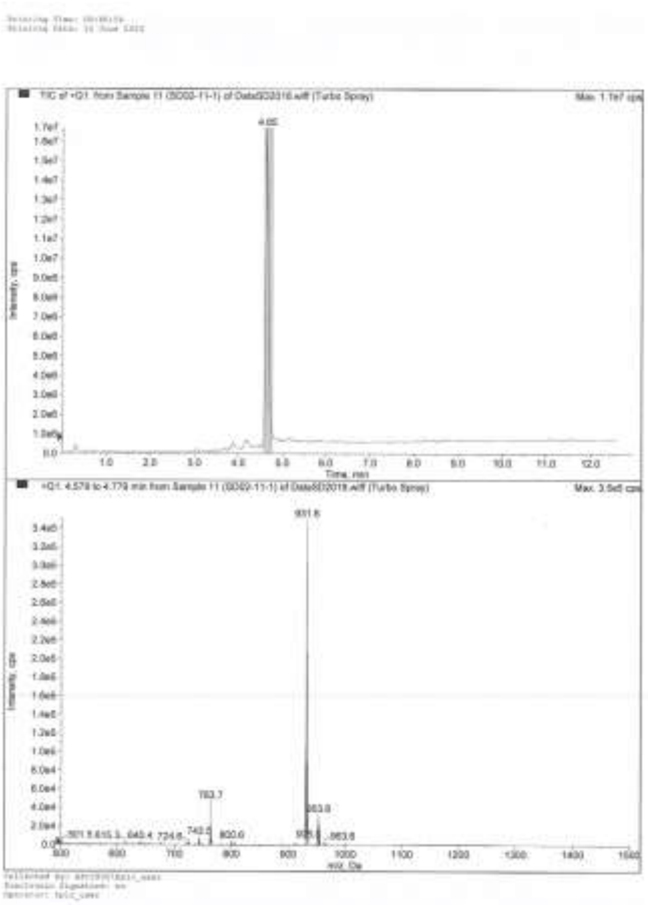

(*R*,*E*)-3-((19-(5,5-difluoro-7,9-dimethyl-5*H*-4λ^4,5λ^4-dipyrrolo[1,2-*c*:2',1'-*f*][1,3,2] diazaborinin-2-yl)-10,17-dioxo-3,6-dioxa-9,16-diazanonadecyl)oxy)-*N*-(3-(2-fluorophenyl)-2-methylallyl)-4,5-dimethoxy-*N*-(2-(1-methylpyrrolidin-2-yl)ethyl) benzamide **(18c):**

Prepared using BODIPY FL-X NHS ester (1 mg, 2.0 pmol) and compound **17c** (2 mg, 2 eq.). LCMS showed complete conversion of the starting materials to the product after stirring overnight (mass found: 975.5; M+H$^+$). Yield: 1.5 mg (77%). Purity of the compound was confirmed to be >95% by LCMS using a long method run.

HRMS calculated for $C_{52}H_{69}BF_3N_6O_8$: 975.5379 [M+H], found: 975.5398 [M+H].

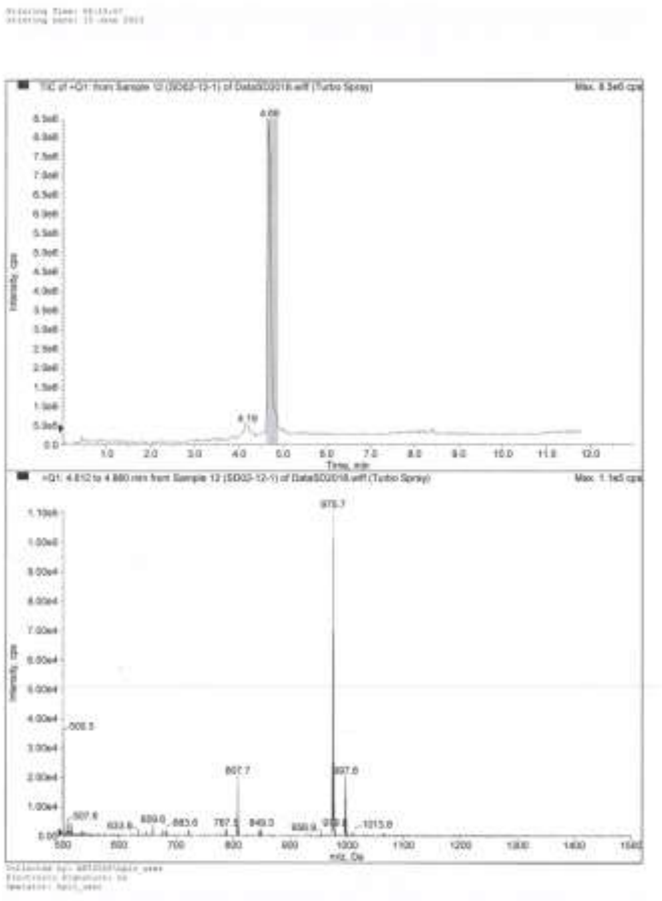

Pharmacology: Material and Methods - no unexpected or unusually high safety hazards were encountered.

All cell culture reagents were purchased from Sigma Chemicals (Poole, Dorset, UK) and plastic were purchased from Sarsted (Nümbrecht, Germany) and Greiner (Stonehouse, UK). Furimazine was purchased from Promega Corporation (Southampton, UK). SNAP-Surface®-Alexa Fluor® 647 was obtained from New England BioLabs (Hitchin UK).

Generation of tagged ACKR3 constructs

The cDNA for human wtACKR3 was provided through the ONCORNET consortium from Vrije Universiteit Amsterdam in the pcDEF3 plasmid. The ACKR3 sequence (with start codon removed) was inserted between the BamH1 and XhoI sites of pcDNA3.1(+) downstream of the SNAP-tag sequence, resulting in a glycine-serine linker between SNAP-tag and ACKR3 sequence. The construction of the NLuc-ACKR3 vector is described in White et al., Cell Chemical Biology, 27(5), 449-510.e7, 2020 (https://doi.org/10.1016/j.chembiol.2020.01.010)

Cell Culture.

The HEK293 Glosensor-NL-ACKR3 cell line was created as described by White et al (2020, as above). All cell lines were cultured in Dulbecco's modified Eagle's medium (DMEM; D6429, Sigma Aldrich), supplemented with 10% FCS. Cells were grown in T75 tissue culture flasks (75 cm^2) in a humidified atmosphere of 95% air and 5% CO_2 at 37°C. Cells were passaged once 70% confluent to prevent loss of protein expression and cell detachment.

NanoBRET Saturation Assays.

HEK293Glosensor-NLuc-ACKR3 cells were seeded into white clear-bottom 96-well plates (Greiner Bio-One 655098 96 well plates), at 40.000 cells/well in DMEM containing 10% FCS and 24 hours prior to the experiment. On the day of experiment, media was removed and cells were washed once with 100 µl of HEPES-Buffered Saline Solution containing 0.2% BSA (HBSS; 145 mM NaCl, 5 mM KCl, 1.3 mM $CaCl_2$, 1 mM $MgSO_4$, 10 mM HEPES, 2 mM sodium pyruvate, 1.5 mM $NaHCO_3$, 10 mM D-glucose, pH 7.45. Cells were then incubated with fluorescent ligand (0-200 nM final concentration) and competing unlabelled ligand (**(R)-1**, final concentration of 10 µM) in a final volume of 50 µL. All compounds were diluted in HBSS/0.2% BSA. The plates were incubated in the dark for 1 hour at 37°C in an incubator with 5% CO_2. The NLuc® substrate furimazine (Promega Corporation) was then added (5 µL per well, 1:400 final concentration) and plates were incubated for 5 minutes then read using the PHERAstar FS plate reader (BMG Labtech, UK) at 37°C. NLuc and BODIPY-FL-X emissions were simultaneously measured using a 475 nm (30 nm band pass filter) and 535 nm (30 nm band pass filter). The resulting raw BRET ratio was calculated by dividing fluorescence emissions by NLuc emissions. Specific binding was calculated by subtracting the non-specific binding from the total binding values.

NanoBRET competition binding assays were performed as above, using a final concentration of 32.4 nM of fluorescent ligand **18c**, added simultaneously with 0-10mM competing ligands and BRET ratios determined as above. The raw BRET ratio was calculated by dividing the fluorescent signal by the bioluminescent signal.

Data Analysis.

All of the data generated was analyzed using Prism 9 software (GraphPad Software, San Diego, USA).

Saturation-binding curves were simultaneously fitted to obtain the total and nonspecific components using the following equation:

$$\text{BRET ratio} = \frac{B_{max}\ x\ [B]}{[B] + K_{\text{D}}} + ((M\ x\ [B]) + C)$$

where Bmax is the maximal level of specific binding, [B] is the concentration of the fluorescent ligand in nM, K_D is the equilibrium dissociation constant in nM, M is the slope of the linear nonspecific binding component, and C is the y-axis intercept. Where the non-specific binding curve was linear, background and non-specific binding components were shared across all datasets with non-specific binding constrained to be greater than 0. When the non-specific binding curves showed saturable binding, total and non-specific binding curves were both fitted to the above equation with only background BRET shared between the data sets. Competition NanoBRET data were fitted using a one-site sigmoidal response curve given by the following equation:

$$\%\ uninhibited\ binding = 100 - \frac{(100\ x\ [A^n])}{([A^n] +\ IC_{50}^n)} +\ NS$$

where [A] is the concentration of competing drug, NS is the nonspecific binding, n is the Hill coefficient, and IC_{50} is the concentration of ligand required to inhibit 50% of the specific binding of the fluorescent ligand. The IC_{50} values from competition-binding curves were used to calculate the Ki of the unlabeled ligands using the Cheng–Prusoff equation:

$$K_i = \frac{IC_{50}}{1 + \frac{[L]}{K_D}}$$

where [L] is the concentration of fluorescent ligand in nM, and K_D is the dissociation constant of the fluorescent ligand in nM. The K_D values used were obtained from the saturation binding experiments.

Confocal microscopy.

For transient transfections, HEK293T cells were seeded at 2 x 10^6 cells/dish onto 28 cm^2 poly-D-lysine coated tissue culture dishes. Cells were transfected overnight with 3 µg pcDNA3.1-SNAP-ACKR3 complexed with PEI (ratio 1:6), then seeded onto poly-D-lysine-coated (10 µg/ml) 8-well Nunc™ Lab-tek™ chambered cover glass (No. 1.0 borosilicate glass bottom) in DMEM supplemented with 10% FCS at a density of 30,000 cells per well. The following day media was replaced with HBSS containing 0.2% BSA and 1 µM SNAP-Surface® Alexa Fluor® 647 (New England Biolabs, Hitchin UK) for 30 min at 37ºC. Cells were then washed in warm HBSS + 0.2 % BSA before pre-incubation in the absence or presence of 10 µM ***(R)*-1** for 30 min at 37°C in a volume of 180 µl HBSS (+0.2% BSA). Fluorescent ligand was then added (20 µl) to each well to achieve a final concentration of 10-100 nM, incubated for 30 min at 37°C and imaged at 37°C.

Cells were imaged on a Zeiss LSM710 (Carl Zeiss, Germany) using a 40x C-Apochromat NA1.2 water immersion objective. Excitation was via 633 nm HeNe (to image SNAP-Surface® Alexa Fluor® 647; SNAP-ACKR3) and 488 nm argon (to image ***(R)-1***) laser lines through a 488/561/633 dichroic and emission collected using a 638-737nm or 493-628nm band pass, respectively. The pinhole was set at 1 Airy unit for the longer wavelength and laser power and gain and offset settings kept constant within experiment to allow comparison. Equatorial plane images were made and 4 images captured per condition per experiment using ZEN 2012 software (Carl Zeiss, Germany) over four independent experiments.

Confocal microscopy.

A time-dependent confocal microscopy study was conducted to study the binding of **18a** and internalization of SNAP-ACKR3.

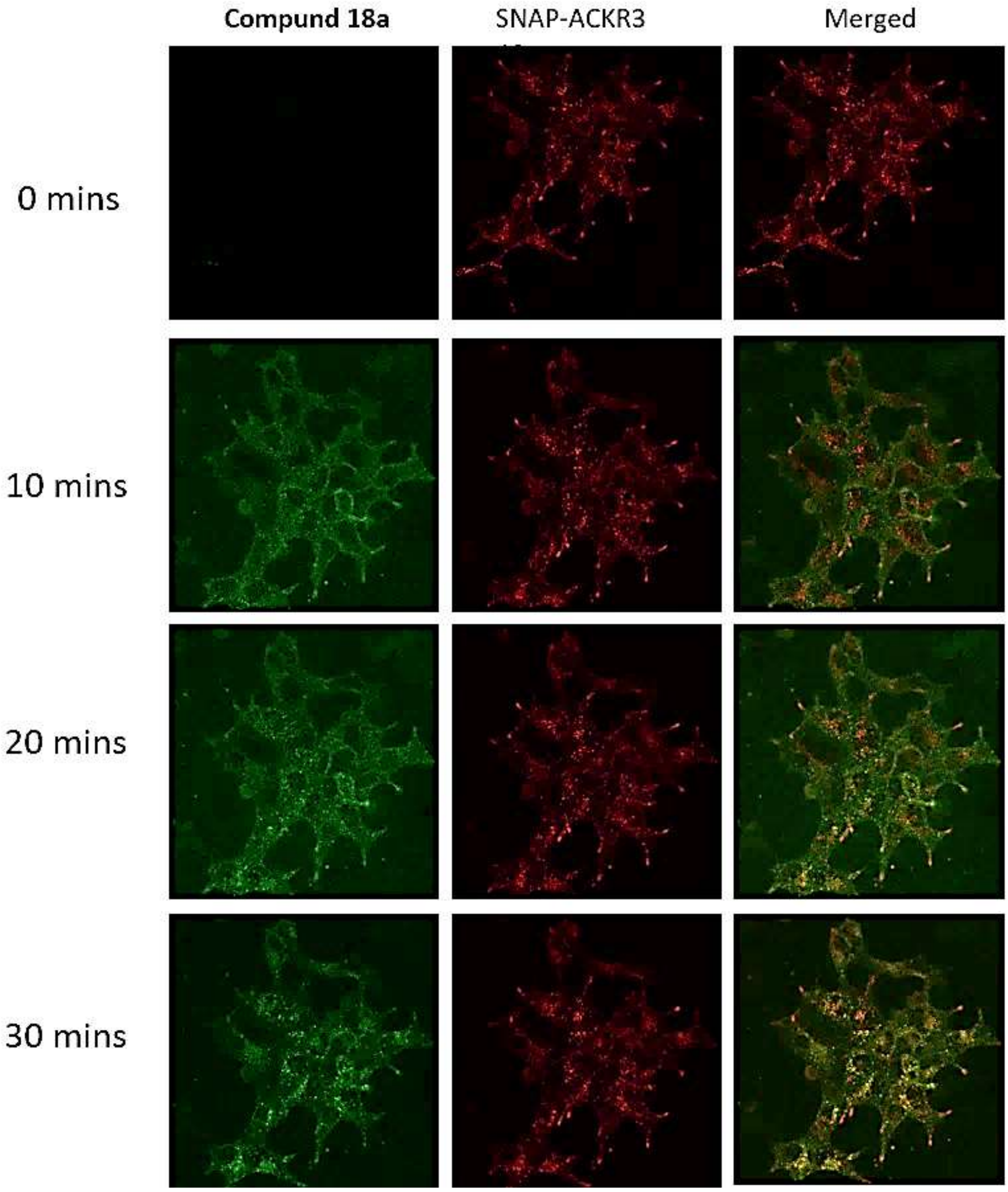

Figure S1: Time-dependent binding of Compound **18a** (50 nM). SNAP-ACKR3 receptor surface expression (red), Compound **18a** (green) overlay image (3rd column). Images were taking on a Zeiss LSM710 confocal microscope, 40x 1.2NA water-immersion objective, following incubation at 37°C for the indicated times. The experiment shown is representative of four independent experiments.

Table S1: Known pharmacological activities of compounds from Figure 1.

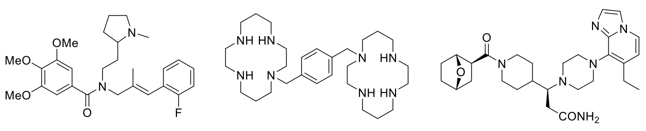

1 VUF11207 **2** AMD3100 **3**

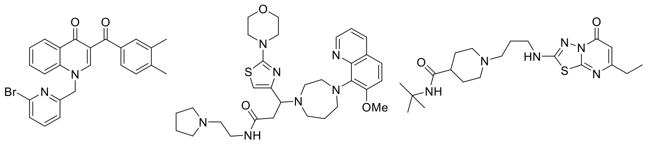

4 **5** CCX777 **6** LN6023

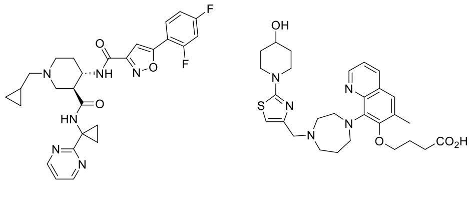

7 ACT-10004-1239 **8** CCX662

Compound Number	Compound Name	Pharmacological activity	Reference
1	VUF11207	Agonist, (pKi = 8.1)<br><br>Induces the recruitment of β-arrestin2 and subsequent interenalisation of CXCR7.<br><br>Measured by competition binding experiments with [^{125}I]-CXCL12 on HEK293 cells stably expressing CXCR7	1
2	AMD3100	Allosteric agonist EC_{50} 140 μM	2
3	N/A	Agonist (pKi = 7.9)<br><br>Measured by competition binding experiments with [^{125}I]-CXCL12 (PerkinElmer) from membranes overexpressing the receptor,<br><br>β-arrestin EC_{50} = 11 nM.<br><br>Measured by CXCR7 β-arrestin-recruitment assay (DiscoverX)	3
4	N/A	Agonist.<br><br>Displaced at least 50% of the SDF-I from the CCXCKR2 receptor at concentrations at or below 100 nM.<br><br>Measured by ^{125}1-labeled SDF-I (Perkin-Elmer Life Sciences) on MCF-7 cells	4, 5
5	CCX777	Partial agonist of β-arrestin-2 recruitment to ACKR3 with an efficacy of 52% and a fourfold	6

Compound Number	Compound Name	Pharmacological activity	Reference
		lower potency (EC_{50} 33 nM versus 8.5) than CXCL12	
6	LN6023	Superagonist of β-arrestin recruitment to ACKR3 of 3.5 μM, efficacy E_{max} 164% *vs* CXCL12. Measured in HEK293T cells using a NanoLuc complementation-based assay (NanoBiT Promega), which measured their ability to induce β-arrestin recruitment to the receptor.	7
7	ACT-1004-1239	Orally available CXCR7 antagonist with an IC_{50} value of 3.2 nM. Measured in a CXCR7-CXCL12 assay using CXCL12 as a ligand.	8
8	CCX662	Inhibits the binding of ^{125}I-CXCL12 to CXCR7 with an IC_{50} of 9 nM, and displays minimal serum shift with an IC_{50} of 18 nM in 100% human serum	9

(1) Wijtmans, M.; Maussang, D.; Sirci, F.; Scholten, D. J.; Canals, M.; Mujić-Delić, A.; Chong, M.; Chatalic, K. L. S.; Custers, H.; Janssen, E.; De Graaf, C.; Smit, M. J.; De Esch, I. J. P.; Leurs, R. Synthesis, Modeling and Functional Activity of Substituted Styrene-Amides as Small-Molecule CXCR7 Agonists. *Eur. J. Med. Chem.* **2012**, *51*, 184–192.

(2) Kalatskaya, I.; Berchiche, Y. A.; Gravel, S.; Limberg, B. J.; Rosenbaum, J. S.; Heveker, N. AMD3100 Is a CXCR7 Ligand with Allosteric Agonist Properties. *Mol. Pharmacol.* **2009**, *75* (5), 1240–1247.

(3) Menhaji-Klotz, E.; Hesp, K. D.; Londregan, A. T.; Kalgutkar, A. S.; Piotrowski, D. W.; Boehm, M.; Song, K.; Ryder, T.; Beaumont, K.; Jones, R. M.; Atkinson, K.; Brown, J. A.; Litchfield, J.; Xiao, J.; Canterbury, D. P.; Burford, K.; Thuma, B. A.; Limberakis, C.; Jiao, W.; Bagley, S. W.; Agarwal, S.; Crowell, D.; Pazdziorko, S.; Ward, J.; Price, D. A.; Clerin, V. Discovery of a Novel Small-Molecule Modulator of C-X-C Chemokine Receptor Type 7 as a Treatment for Cardiac Fibrosis. *J. Med. Chem.* **2018**, *61* (8), 3685–3696.

(4) Uto-Konomi, A.; McKibben, B.; Wirtz, J.; Sato, Y.; Takano, A.; Nanki, T.; Suzuki, S. CXCR7 Agonists Inhibit the Function of CXCL12 by Down-Regulation of CXCR4. *Biochem. Biophys. Res. Commun.* **2013**, *431* (4), 772–776.

(5) Anita Melikian; John Jessen Wright; Antoni Krasinski; Cheng Hu; Aaron Novack. Substituted Quinolones and Methods of Use. WO2007059108A2, 2007.

(6) Gustavsson, M.; Wang, L.; van Gils, N.; Stephens, B. S.; Zhang, P.; Schall, T. J.; Yang, S.; Abagyan, R.; Chance, M. R.; Kufareva, I.; Handel, T. M. Structural Basis of Ligand Interaction with Atypical Chemokine Receptor 3. *Nat. Commun.* **2017**, *8* (1), 14135.

(7) Bayrak, A.; Mohr, F.; Kolb, K.; Szpakowska, M.; Shevchenko, E.; Dicenta, V.; Rohlfing, A. K.; Kudolo, M.; Pantsar, T.; Günther, M.; Kaczor, A. A.; Poso, A.; Chevigné, A.; Pillaiyar, T.; Gawaz, M.; Laufer, S. A. Discovery and Development of First-in-Class ACKR3/CXCR7 Superagonists for Platelet Degranulation Modulation. *J. Med. Chem.* **2022**, *65* (19), 13365–13384.

(8) Richard-Bildstein, S.; Aissaoui, H.; Pothier, J.; Schäfer, G.; Gnerre, C.; Lindenberg, E.; Lehembre, F.; Pouzol, L.; Guerry, P. Discovery of the Potent, Selective, Orally Available CXCR7 Antagonist ACT-1004-1239. *J. Med. Chem.* **2020**, *63* (24), 15864–15882.

(9) Yen, Y.-C.; Schafer, C. T.; Gustavsson, M.; Eberle, S. A.; Dominik, P. K.; Deneka, D.; Zhang, P.; Schall, T. J.; Kossiakoff, A. A.; Tesmer, J. J. G.; Handel, T. M. Structures of Atypical Chemokine Receptor 3 Reveal the Basis for Its Promiscuity and Signaling Bias. *Sci. Adv.* **2022**, *8* (28).
